# Myosin-I’s motor and actin assembly activation activities are modular and separable in budding yeast clathrin-mediated endocytosis

**DOI:** 10.17912/micropub.biology.001223

**Published:** 2024-06-04

**Authors:** Jennifer M Hill, Ross TA Pedersen, David G Drubin

**Affiliations:** 1 Molecular and Cell Biology, University of California, Berkeley, Berkeley, CA, United States

## Abstract

The myosin-Is, Myo3 and Myo5 in budding yeast, are implicated in force generation and actin assembly during clathrin-mediated endocytosis (CME). The myosin-Is have motor activity, bind the plasma membrane, and activate the Arp2/3 complex to promote branched actin assembly. We reveal that Myo5 's force-generating motor activity and nucleation-promoting factor (NPF) activity each must be coupled to membrane binding for successful CME. However, the motor and NPF activities are modular and separable, showing that these activities function independently rather than in an obligatorily integrated manner to provide myosin-I's essential functions in actin network assembly and force generation during budding yeast CME.

**Figure 1. Myosin-I motor and SH3 domain mutants genetically complement to rescue endocytic internalization and dynamics.**
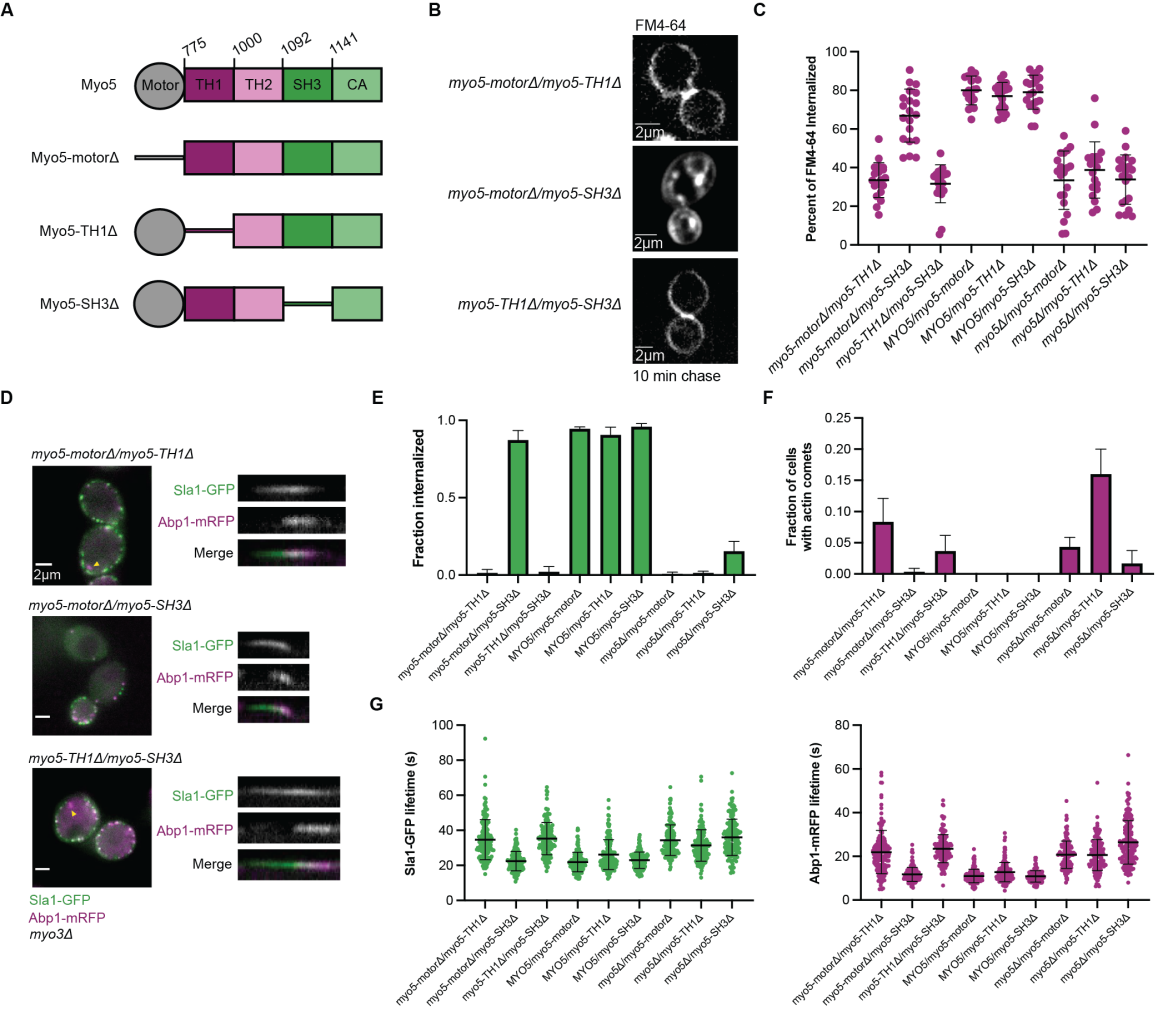
(A) Myosin-I domain structure and mutants. TH1: Tail homology 1, TH2: Tail homology 2, SH3: Src homology 3, CA: Central acidic. (B) Representative images of FM4-64 signal at the medial plane of cells 10 min post-dye chase. (C) Percent of FM4-64 internalized by myosin-I complementation strains 10 min post-dye chase (n=20 cells). Error bars show standard deviation. (D) Representative images of Sla1-GFP and Abp1-mRFP in myosin-I complementation strains and representative kymographs of individual endocytic events in these strains. Yellow arrows indicate endocytic sites with actin comets. (E) Fraction of endocytic sites that internalize in heterozygous myosin-I mutant diploid strains. Sites where Sla1 and Abp1 hook into the cell were considered internalized (n=150 sites from ≥15 cells). (F) Fraction of cells with actin comets in heterozygous myosin-I mutant diploid strains (n= 150 cells). (G) Sla1-GFP (left) and Abp1-mRFP (right) lifetimes at endocytic sites in myosin-I complementation strains (n=150 sites from ≥15 cells). Error bars show standard deviation.

## Description

Budding yeast CME occurs under high turgor pressure and, therefore, requires the forces produced by the assembly of a branched actin network and actin-associated proteins (Aghamohammadzadeh & Ayscough, 2009; Sun et al., 2006) . Myosins are an important contributor to this process both as actin-associated molecular motors and as actin assembly factors (Evangelista et al., 2000; Geli et al., 2000; Jonsdottir & Li, 2004; Pedersen et al., 2023; Sun et al., 2006) . The myosins involved in CME are the type I myosins, a class of motors in the myosin family (Geli & Riezman, 1996; McIntosh & Ostap, 2016) . In wild-type cells, the myosin-Is localize to the base of the endocytic invagination where they act as an indispensable connection between actin filaments and the plasma membrane (Pedersen & Drubin, 2019) . They are also nucleation-promoting factors (NPF's), activating the Arp2/3 complex to generate a branched actin network (Evangelista et al., 2000; Geli et al., 2000; Sun et al., 2006) . Cells lacking both myosins build branched actin networks at endocytic sites, but these networks are insufficient for driving vesicle internalization (Geli & Riezman, 1996; Sun et al., 2006) . Despite their requirement for CME, the exact mechanism of myosin-I function is still incompletely understood.

Myo5 and Myo3 are long-tailed myosin-Is, harboring several domains in the tail region that contribute to overall function of the myosin (Lewellyn et al., 2015) ( Fig. 1A ). The tail homology 1 (TH1) domain binds plasma membrane phospholipids. It is necessary for the proper recruitment of myosin-I to endocytic sites, and anchors actin assembly to the membrane, a myosin-I CME function (Pedersen & Drubin, 2019) . Located C-terminal to the TH1 domain is a Src Homology 3 (SH3) domain that also contributes to proper myosin-I recruitment to CME sites (Lewellyn et al., 2015) and, in concert with the central acidic (CA) domain, is required for activation of the Arp2/3 complex to promote branched actin assembly (Evangelista et al., 2000; Geli et al., 2000; Sun et al., 2006) . Additionally, Myo5 contains a motor domain that is a low-duty-ratio motor with force-insensitive binding and unbinding kinetics *in vitro* , indicating that it is likely to be a power-generating motor, capable of assisting actin assembly in generating force for vesicle internalization (Pedersen et al., 2023) and enabling actin assembly (Manenschijn et al., 2019) . Given the highly modular domain structure of myosin-I in budding yeast and the wide variety of roles and interactions attributed to the individual domains of the protein, there is much to be learned about the interplay between the functional modules of myosin-I in CME.

To better understand the functional relationships between the myosin-I motor, SH3, and TH1 domains, we asked whether the activities provided by each domain need to function on the same myosin molecule in order to achieve the essential CME function of the protein. We generated a set of strains to test for intragenic complementation among different combinations of the Myo5 domain mutants: *myo5-TH1Δ/myo5-motorΔ* , *myo5-motorΔ/myo5-SH3Δ* , and *myo5-TH1Δ/myo5-SH3Δ* , in a *myo3∆* background, since Myo5 and Myo3 behave in a genetically redundant manner with respect to the CME functions. Additionally, we generated diploid strains expressing a combination of each Myo5 domain mutant and the wild-type *MYO5* allele or a *myo5∆* allele.

To determine whether the myosin-I domain mutant combinations can support internalization of vesicles by CME, we pulse-labeled cells with the membrane dye FM4-64 and quantified internalization of the membrane dye following a 10-minute chase ( Fig. 1B and Fig . 1C). Strains with a wild-type *MYO5* allele exhibited FM4-64 staining on internal membrane structures, having internalized ~80% of the dye from the plasma membrane ( Fig. 1C ). In contrast, strains with only a *myo5∆* allele exhibited negligible staining of internal membranes, with only ~35% of the dye internalized from the plasma membrane ( Fig. 1C ), indicating that endocytosis is defective in these strains. The *myo5-TH1Δ/myo5-motorΔ* and *myo5-TH1Δ/myo5-SH3Δ* cells only internalized 33.5% and 31.6% of the FM4-64 from the plasma membrane, respectively ( Fig. 1B and Fig . 1C), indicating that these myosin domain mutants do not complement to rescue FM4-64 internalization, and that the membrane binding domain must be on the same molecule as the motor domain and SH3 domain. FM4-64 behavior in the *myo5-motorΔ/myo5-SH3Δ* cells, however, resembled that of strains complemented with a wild-type *MYO5* allele, with 90.5% of the dye internalized from the plasma membrane ( Fig. 1B and Fig . 1C). This result indicates that the *myo5-motorΔ* and *myo5-SH3Δ* alleles functionally complement in the context of substrate internalization and shows that myosin-I motor and NPF activities do not need to be on the same myosin molecule for successful CME.

Next, we investigated CME protein dynamics in the heterozygous myosin domain mutant strains. To track endocytic internalization, we used a fluorescent fusion of the early coat protein Sla1 as a marker for the endocytic coat, and a fusion of the actin-binding protein Abp1 as a marker for the actin network ( Fig. 1D ). Using Sla1-GFP as a marker for internalization, the fraction of CME sites at which these fluorescent marker proteins internalize in diploid strains with a wild-type *MYO5* allele is ~0.95, while the internalized fraction in diploid strains with a *myo5∆* allele is ~0.1, similar to the internalized fractions in wild-type cells and *myo5∆* haploid cells, respectively (Lewellyn et al., 2015; Sun et al., 2006) ( Fig. 1E ). The success rate of CME in the *myo5-TH1Δ/myo5-motorΔ* and *myo5-TH1Δ/myo5-SH3Δ* cells is low, similar to that of strains with a *myo5∆* allele ( Fig. 1D and Fig . 1E), and kymographs of individual endocytic events show that Sla1-GFP and Abp1-mRFP remain at the plasma membrane, indicative of failed CME. However, kymographs of individual endocytic events in *myo5-motorΔ/myo5-SH3Δ* cells show that Sla1-GFP and Abp1-mRFP hook into the cell at the end of the event, with an internalized fraction of 0.87, similar to that of strains with a fully wild-type *MYO5* allele ( Fig. 1D and Fig . 1E). Thus, the *myo5-motorΔ* and *myo5-SH3Δ* alleles are capable of complementing to support functional endocytic internalization, while neither of the *myo5-TH1Δ* combinations are able to support endocytic internalization.

A common phenotype in cells expressing Myo5 lacking a TH1 domain is the formation of actin comets associated with the plasma membrane at CME sites, indicative of an actin network incapable of harnessing the force generated by actin polymerization to drive vesicle internalization (Pedersen & Drubin, 2019) . To test whether the myosin domain mutant combinations displayed this phenotype, we counted the fraction of cells that developed actin comets, read out by Abp1-mRFP structure, throughout a two-minute movie. Actin comets were never observed in cells containing a functional *MYO5* allele, and were observed in ~16% of the cells containing only Myo5-TH1Δ ( *myo5-TH1Δ/myo5Δ* cells) ( Fig. 1F ), corroborating previous results using haploid cells (Pedersen & Drubin, 2019) . In contrast, actin comets were only observed in 0.3% of *myo5-motorΔ/myo5-SH3Δ* cells ( Fig. 1F ). However, for both myosin domain mutant combinations containing a *myo5-TH1Δ* , actin comets were observed at a significantly higher frequency than in strains expressing a wild-type *MYO5* allele ( Fig. 1F ), indicating that actin comets are a dominant phenotype caused by Myo5 lacking a TH1 domain.

CME protein lifetimes can act as a secondary readout for CME success, so we measured the lifetimes of Sla1 and Abp1. In cells expressing a wild-type *MYO5* allele, Sla1 and Abp1 lifetimes resembled those in haploid wild-type cells, while in cells carrying a *myo5Δ* allele, Sla1 and Abp1 had increased lifetimes, similar to those in haploid *myo5Δ* cells (Kaksonen et al., 2005; Sun et al., 2006) ( Fig. 1G ). Sla1 and Abp1 lifetimes in the *myo5-TH1Δ/myo5-motorΔ* and *myo5-TH1Δ/myo5-SH3Δ* cells were also significantly higher, again indicating a CME defect and suggesting that these mutant myosin combinations are not able to support robust CME dynamics. The *myo5-motorΔ/myo5-SH3Δ* cells, however, had Sla1 and Abp1 lifetimes that were similar to those in strains expressing the wild-type *MYO5* allele ( Fig. 1G ), indicating that Myo5-motorΔ and Myo5-SH3Δ proteins combined are able to support nearly normal endocytic dynamics.

Taken together, these data show that the yeast type I myosin motor and SH3 domain mutants can complement to rescue membrane internalization and endocytic dynamics phenotypes, demonstrating that successful CME does not require motor and NPF activity to be present in the same molecule. Thus, motor activity and Arp2/3 complex activation are functionally separable roles that must both be present in the same actin network, but not in the same myosin molecule. In contrast, in cells expressing both the myosin TH1 deletion mutant and either the motor mutant or the SH3 mutant, successful CME is not supported. This result demonstrates that the myosin-I membrane binding domain must be on the same molecule with a functioning motor or SH3 domain for those domains to support successful CME. These results establish that the TH1 domain plays a crucial role in localizing both motor activity and actin polymerization activity to the plasma membrane. Endocytic proteins tend to be highly multi-valent, and the domains are generally conserved, though their specific arrangements within proteins can vary (Dergai et al., 2016; Elde et al., 2005; Macro et al., 2012) . It appears that within the complex network of interacting CME proteins, the presence of these domains at the plasma membrane and in the endocytic network is, at least in some cases, more important than how those domains are situated in proteins.

## Methods

**Strains and Plasmids**

All budding yeast strains were maintained in standard rich media (YPD) at 25°C since *myo5Δ* and domain mutant strains are temperature sensitive. The strains used in this study were derived from the wild-type diploid strain DDY1102 using standard techniques and are listed in Table 1.

**Live-Cell Imaging**

Cells were grown to mid-log phase in imaging media (synthetic minimal media supplemented with adenine, l-histidine, l-leucine, l-lysine, l-methionine, uracil, and 2% glucose) at 25°C and adhered to coverslips coated with 0.2 mg/ml concanavalin A.

For FM4-64 pulse-chase experiments, cells were equilibrated with a quick wash of imaging media with 10 µg/ml FM4-64 (Molecular Probes), and then incubated for 5 min at room temperature in imaging media with 10 µg/ml FM4-64. Samples were then washed vigorously with fresh imaging media to remove excess FM4-64. Cells were imaged at room temperature after a 10 min chase on a Nikon Eclipse Ti inverted microscope with a Nikon 100× 1.45-NA Plan Apo λ oil immersion objective using Nikon Elements software. Images were acquired using an Andor IXon X3 EM-CCD camera, and an Andor CSU-X spinning disc confocal setup. FM4-64 was excited using a 488-nm laser and a 500 ms exposure time, and detected with a Chroma 605/52-nm emission filter.

Epifluorescence microscopy of Sla1-GFP and Abp1-mRFP was performed using a Nikon Eclipse Ti inverted microscope with a Nikon 100× 1.4-NA Plan Apo VC oil-immersion objective and an Andor Neo 5.5 sCMOS camera using Nikon Elements software. Coverslips were imaged in an environmental chamber (In Vivo Scientific) pre-warmed and maintained at 25°C. Two color movies of the medial focal plane were acquired sequentially for 2 min using an FF-493/574-Di01 dual-pass dichroic mirror and FF01-512/630-25 dual-pass emission filters (Semrock). GFP and RFP were excited with a Lumencore Spectra X LED light source with 550/515-nm and 470/422-nm excitation filters with 500 ms exposure times for each channel.

**Image Analysis**

All image analysis was performed using Fiji software (National Institutes of Health). All images and movies were subjected to background subtraction and photobleaching correction (Kaksonen et al., 2003) . For FM4-64 pulse-chase experiments, cells that were stained brightly for FM4-64 were removed from the analysis as they were considered to be dead or to have compromised plasma membrane permeability. The percent of FM4-64 internalized was calculated by dividing the integrated FM4-64 fluorescence signal inside of the plasma membrane by the integrated FM4-64 fluorescence signal of the entire cell.

For quantification of patch internalization and Sla1-GFP and Abp1-mRFP lifetimes, radial kymographs were generated from cells and chosen at random for quantitative analysis. A patch was judged as “internalized” if it moved at least 200 nm toward the cell interior. A patch was judged as “failed” if it did not move toward the cell interior, moved fewer than 200 nm toward the cell interior, or moved toward the cell interior before returning to its original position.

## Extended Data

Description: Strains used in this study. Resource Type: Collection. DOI: 10.22002/0414a-zvs53

## References

[R1] Aghamohammadzadeh S, Ayscough KR (2009). Differential requirements for actin during yeast and mammalian endocytosis.. Nat Cell Biol.

[R2] Dergai M, Iershov A, Novokhatska O, Pankivskyi S, Rynditch A (2016). Evolutionary Changes on the Way to Clathrin-Mediated Endocytosis in Animals.. Genome Biol Evol.

[R3] Elde NC, Morgan G, Winey M, Sperling L, Turkewitz AP (2005). Elucidation of clathrin-mediated endocytosis in tetrahymena reveals an evolutionarily convergent recruitment of dynamin.. PLoS Genet.

[R4] Evangelista M, Klebl BM, Tong AH, Webb BA, Leeuw T, Leberer E, Whiteway M, Thomas DY, Boone C (2000). A role for myosin-I in actin assembly through interactions with Vrp1p, Bee1p, and the Arp2/3 complex.. J Cell Biol.

[R5] Geli MI, Lombardi R, Schmelzl B, Riezman H (2000). An intact SH3 domain is required for myosin I-induced actin polymerization.. EMBO J.

[R6] Geli MI, Riezman H (1996). Role of type I myosins in receptor-mediated endocytosis in yeast.. Science.

[R7] Jonsdottir GA, Li R (2004). Dynamics of yeast Myosin I: evidence for a possible role in scission of endocytic vesicles.. Curr Biol.

[R8] Kaksonen M, Sun Y, Drubin DG (2003). A pathway for association of receptors, adaptors, and actin during endocytic internalization.. Cell.

[R9] Kaksonen M, Toret CP, Drubin DG (2005). A modular design for the clathrin- and actin-mediated endocytosis machinery.. Cell.

[R10] Lewellyn EB, Pedersen RT, Hong J, Lu R, Morrison HM, Drubin DG (2015). An Engineered Minimal WASP-Myosin Fusion Protein Reveals Essential Functions for Endocytosis.. Dev Cell.

[R11] Macro L, Jaiswal JK, Simon SM (2012). Dynamics of clathrin-mediated endocytosis and its requirement for organelle biogenesis in Dictyostelium.. J Cell Sci.

[R12] Manenschijn HE, Picco A, Mund M, Rivier-Cordey AS, Ries J, Kaksonen M (2019). Type-I myosins promote actin polymerization to drive membrane bending in endocytosis.. Elife.

[R13] McIntosh BB, Ostap EM (2016). Myosin-I molecular motors at a glance.. J Cell Sci.

[R14] Pedersen RTA, Drubin DG (2019). Type I myosins anchor actin assembly to the plasma membrane during clathrin-mediated endocytosis.. J Cell Biol.

[R15] Pedersen RTA, Snoberger A, Pyrpassopoulos S, Safer D, Drubin DG, Ostap EM (2023). Endocytic myosin-1 is a force-insensitive, power-generating motor.. J Cell Biol.

[R16] Sun Y, Martin AC, Drubin DG (2006). Endocytic internalization in budding yeast requires coordinated actin nucleation and myosin motor activity.. Dev Cell.

